# Influence of Air Humidity Level on the Structure and Mechanical Properties of Thermoplastic Starch-Montmorillonite Nanocomposite during Storage

**DOI:** 10.3390/ma16030900

**Published:** 2023-01-17

**Authors:** Natália Šmídová, Hamed Peidayesh, Anton Baran, Oľga Fričová, Mária Kovaľaková, Ružena Králiková, Ivan Chodák

**Affiliations:** 1 Department of Physics, Faculty of Electrical Engineering and Informatics, Technical University of Košice, Park Komenského 2, 042 00 Košice, Slovakia; 2 Polymer Institute, Slovak Academy of Sciences, Dúbravská cesta 9, 845 41 Bratislava, Slovakia; 3 Department of Environmental Engineering, Faculty of Mechanical Engineering, Technical University of Košice, Park Komenského 5, 042 00 Košice, Slovakia

**Keywords:** thermoplastic starch nanocomposite, montmorillonite, relative humidity, NMR, mechanical properties

## Abstract

Thermoplastic starch (TPS) consisting of corn starch and glycerol as a plasticizer, and TPS-montmorillonite (MMT) nanocomposite were stored at room temperature in the air with relative humidities (RH) of 11, 55 and 85% for seven weeks. Mechanical testing and dynamic mechanical thermal analysis (DMTA) were performed to detect changes in their mechanical properties. Solid-state NMR spectroscopy monitoring the changes in molecular mobility in the samples provided an insight into relations between mechanical properties and local structure. The results of mechanical testing indicated that the addition of MMT results in the increase in the tensile strength and Young’s modulus while elongation at break decreased, indicating the reinforcing effect of MMT. DMTA experiments revealed a decrease in glass transition temperature of starch-rich phase below room temperature for samples stored at higher RH (55 and 85%). This indicates that absorbed water molecules had additional plasticizing effect on starch resulting in higher mobility of starch chain segments. Recrystallization in these samples was deduced from the shape of cross-polarization magic-angle spinning ^13^C NMR spectra. The shape of broad-line ^1^H NMR spectra reflected changes in molecular mobility in the studied samples during seven weeks of storage and revealed that a high amount of water molecules impacts the starch intermolecular hydrogen bond density.

## 1. Introduction

Thermoplastic starch (TPS) is generally prepared by the addition of plasticizers into the starch matrix using a thermomechanical process. An organic low molecular weight compound called plasticizer penetrates the starch molecules and creates hydrogen bonds with the hydroxyl groups of starch, resulting in an increase in free volume and consequently an increase in molecular mobility [1,2]. TPS-based materials have been considered in the biopolymer industry due to their biodegradability, availability from renewable resources, and low cost [3,4,5]. However, TPS suffers from recrystallization (retrogradation) and cocrystallization of amylose chains caused by humidity and its hydrophilic character. This evolution leads to substantial negative changes in mechanical properties during storage [6,7]. From this point of view, in many cases the addition of a small amount of nanofillers improves substantially the physico-chemical properties of TPS-based materials [8,9].

As the main groups of nanofillers, the layered silicates are frequently used for the preparation of polymer nanocomposites. Clay is considered to be an important mineral among the layered silicates, with a high amount of water molecules in the silicate layer. Most of the clays are structurally and chemically unique and may absorb various amounts of water that may affect the process of cations replacement [10,11,12]. Recently, a large number of studies are focused on montmorillonite (MMT) due to its well-controlled chemical properties, availability, and versatility towards the environment and health [13,14]. The surface chemistry of silicate through ion-exchange reactions and their ability to disperse in layers are the most important MMT properties that significantly affect the mechanical properties, thermal stability, and water resistance of the nanocomposite product. Hence, the hydration of Na^+^ and K^+^ ions occurring in the clays allows the clays to swell and provides the ability for polymer chains to intercalate between the layers [15] or under suitable conditions even exfoliation of the MMT may occur [16,17]. The ultimate properties of TPS-MMT nanocomposites are closely related to the arrangement of starch chains, molecular dynamics, and the dispersion of the clay fillers within the TPS matrix [18].

In designing TPS for a given purpose, mutual correlations between the source of starch, method of its modification, processing technique, physical and material parameters, as well as performance in various conditions should be considered. It is well known that TPS-based materials are sensitive to the surrounding relative humidity [19] and storage time, which substantially affect the polymer structure and molecular mobility and subsequently their mechanical properties. Investigating the structure and molecular mobility of TPS-based materials during aging is described in the literature [6,20,21,22]. However, to the best of our knowledge, no investigation has been done to evaluate the influence of aging under exactly defined relative humidities on the molecular mobility of TPS-MMT nanocomposite.

The main aim of the present work was to find the correlation between the key parameters of the structure of the TPS-MMT nanocomposite and performance properties according to the determined application. In this regard, changes in the starch chain arrangements and nanofiller particles in TPS-MMT nanocomposite as well as their mechanical properties were evaluated using solid-state ^1^H and ^13^C NMR spectroscopy, tensile testing and dynamic mechanical thermal analysis (DMTA).

## 2. Materials and Methods

### 2.1. Materials

Native corn starch Meritena^®^ 100 was provided by Brenntag (Bratislava, Slovakia). Its water content was around 12 wt% as determined by drying in the oven at 105 °C for 5 h. Natrified montmorillonite (Cloisite^®^ Na^+^) with a cationic exchange capacity range of 80–95 mequiv/100 g was purchased from Southern Clay Products (Texas, Gonzales, USA). Glycerol was obtained from Centralchem, Ltd. (Bratislava, Slovakia). Double distilled water was used for the preparation of all solutions.

### 2.2. Preparation of TPS and TPS-MMT Nanocomposite Samples

The composition of the mixture consisted of 1 part of starch (dried), 0.5 parts of glycerol, and 2.3 parts of distilled water with or without dispersed Cloisite Na (MMT). To prepare nanocomposite samples, 0.02 parts (parts based on dry weight of starch) of MMT nanoparticles were dispersed in water and glycerol mixture for 5 min by mechanical mixing. Afterward, the starch was added to the prepared mixture and the suspension was annealed at 80 °C for 10 min under continuous mixing for complete gelatinization. The obtained mixtures were heated in an oven at 100 °C for 5 h followed by maintaining overnight at 60 °C to prevent moisture absorption. Then, the dried material was mixed in a laboratory mixer Plastograph Brabender PLE331 for 10 min at 130 °C and 100 rpm. Slabs 1 mm thick were prepared by compression molding (laboratory press, LabEcon 300) at 130 °C applying 2 min preheating without pressure and an additional 2 min at a pressure of 2.65 MPa.

The TPS and TPS-MMT samples were stored in dessicators at different relative humidities (RH) namely 11, 55 and 85% using saturated salt solutions at 25 °C with lithium chloride (LiCl), magnesium nitrate (Mg(No_3_)_2_), and potassium chloride (KCl) [23], respectively. In the next text, storage at particular RH is indicated by the number, e.g., nanocomposite stored at 55% RH is denoted as TPS-MMT55.

### 2.3. Tensile Testing

The compression-molded slabs were punched by pneumatic toggle press equipment to prepare dumbbell-shaped test specimens with a 3.5 mm × 30 mm dimension of the deformed area during the tensile test (thickness of each specimen of approximately 1 mm was exactly measured before testing to be used for strength and modulus calculations). The tensile properties were measured by using an Instron 3365 (Instron, Norwood, MA, USA) universal testing machine in uniaxial deformation at a cross-head speed of 1 mm/min up to 1% deformation (to determine Young’s modulus) followed by a speed of 50 mm/min at higher deformations. The average values and standard deviations were calculated from seven specimens for all parameters.

### 2.4. Dynamic Mechanical Thermal Analysis (DMTA)

Dynamic-mechanical measurements were performed using the instrument DMA Q800 (TA Instruments, Hüllhorst, Germany). The specimens (ca. 10 mm × 7 mm × 1 mm) were measured in tensile mode at a frequency of 10 Hz and amplitude of dynamic deformation of 20 μm. The experiments were performed in the temperature range from −70 °C to 140 °C at a heating rate of 2 °C/min.

### 2.5. Water Content

First, the weight of TPS and TPS-MMT specimens (*W*_1_) was measured after drying at 80 °C for 4 h and was taken as the weight of dry specimen. Then, the samples in triplicate were placed in desiccators with different relative humidities and weighed (*W*_2_) after various periods. The total water content, *W*, in percent was calculated according to the equation below:

(1) $$W\;\left(\%\right)=\frac{W_{2}-W_{1}\;}{W_{2}}\times 100$$

Dependence of the water content of the particular samples on the storage time will be discussed in Section 3.4.

### 2.6. ^1^H and ^13^C NMR Measurements

All NMR experiments were performed on a Varian solid-state NMR spectrometer (Palo Alto, CA, USA) equipped with a probe head with 4 mm ZrO_2_ rotors at ambient temperature. ^13^C and ^1^H resonance frequencies were approximately 100 and 400 MHz, respectively.

The high-resolution ^13^C NMR spectra were recorded using the cross-polarization technique (CP) and magic angle spinning (MAS) rate of 10 kHz. The measurements were performed with the radio frequency field strength of 63 kHz in accordance with Hartmann–Hahn condition. CP contact time 1 ms, acquisition time 40 ms, and relaxation delay between two consecutive scans 7 s were used. SPINAL pulse high-power proton decoupling of 63 kHz was applied. The number of scans was 10,000.

Broad-line (BL) ^1^H NMR spectra were acquired using Chen sequence suppressing the broad probehead background, the ^1^H MAS NMR spectra were accumulated at the spinning rate of 10 kHz using single pulse sequence. The same experimental conditions were applied during accumulation of both BL and MAS ^1^H NMR spectra: π/2 pulse with duration 3.8 μs and 10 s recycle delay. Acquisition times 60 and 150 ms were used at measurement of BL and MAS ^1^H NMR spectra, respectively.

The chemical shifts of all spectra were referenced to tetramethyl silane using adamantane as an external standard.

## 3. Results

### 3.1. ^13^C CP MAS NMR

**Figure 1:**
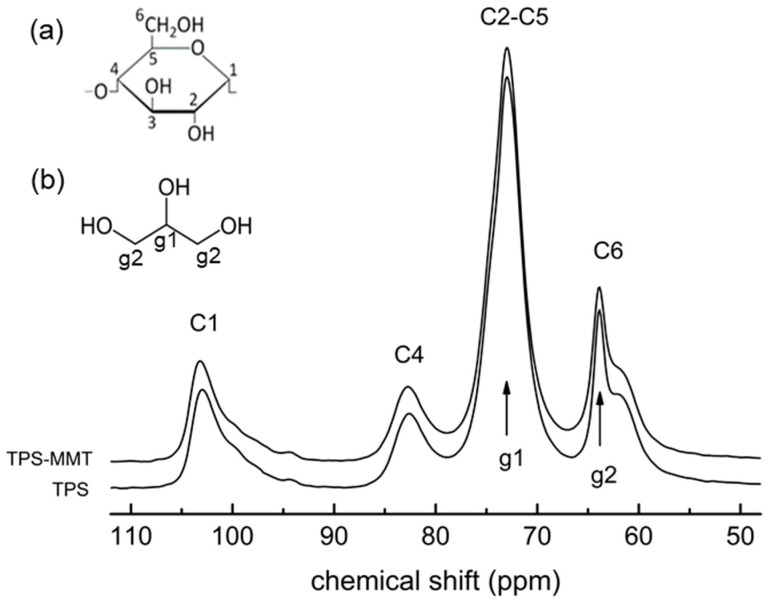
^13^C CP MAS NMR spectra measured for dried TPS and TPS-MMT samples. The C1–C6 and g1, g2 resonances relate to the carbons of starch monomer unit and glycerol, respectively. Chemical structures of (**a**) thermoplastic starch monomer unit (*α*-D-glucose) and (**b**) glycerol are depicted in the inset.

The ^13^C CP MAS NMR spectra for both dried TPS and TPS-MMT samples are almost identical (Figure 1). The resonances in the spectra are related to the carbons of starch and glycerol (chemical structures shown in Figure 1) as follows: 103 ppm—starch C1 carbon, 82 ppm—starch C4 carbon in amorphous structure, around 73 ppm—starch C2, C3, C5 carbons and C4 carbon in ordered structure, 63 ppm—starch C6 carbon, signals 73 and 64 ppm—glycerol carbons. The shape of C1 carbon resonance indicates that the structure of both samples is fully amorphous [24,25,26].

**Figure 2:**
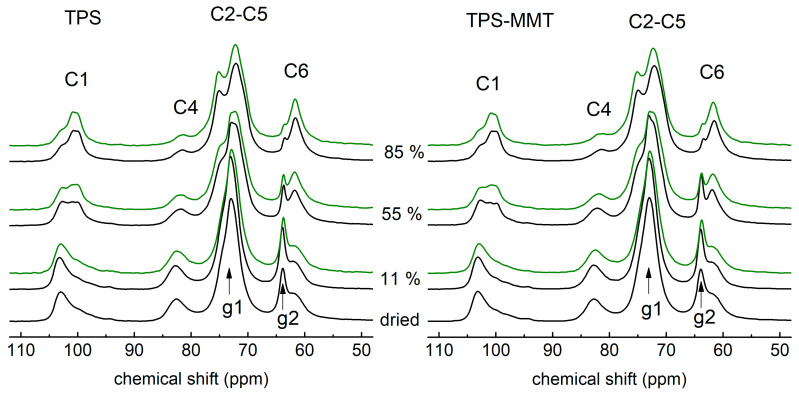
^13^C CP MAS NMR spectra measured for TPS (**left**) and TPS-MMT (**right**) samples dried and stored at indicated RHs for one week (black) and seven weeks (green). The C1–C6 and g1, g2 resonances relate to the carbons of starch monomer unit and glycerol, respectively.

The spectra for TPS and TPS-MMT samples measured after one and seven weeks of storage at 11% RH are similar to the spectra of the dried samples (Figure 2). Some differences can be observed in the intensities of the glycerol signal at 62 ppm and the signal at 73 ppm which is a superposition of starch C2–C5 and glycerol COH resonances. Since the polarization transfer between ^1^H and ^13^C nuclei is restricted by their enhanced relative motion, a small decrease in the intensities of 62 and 73 ppm signals could be caused by increased mobility of glycerol molecules due to physical ageing of the samples during storage. Low water content does not significantly affect the starch chains mobility; it prevents the starch recrystallization and thus the samples remain fully amorphous during storage similarly as dried samples.

The spectra measured for TPS and TPS-MMT samples stored at 55 and 85% RH differ significantly from the spectra of the samples, both dried as well as stored at relative humidity of 11% (Figure 2). The shape of C1 signal indicates that ordered (crystalline) starch structure of B-type [24,25,26] was formed in these samples during the first week of storage. This change is accompanied by the decrease in the intensity of C4 signal and by narrowing of the C6 signal due to more enhanced polarization transfer between ^1^H and ^13^C nuclei present in ordered structure. These differences are the most pronounced in the spectra for TPS85 and TPS-MMT85 samples with the highest water content. In these spectra the superimposed signal of C2–C5 starch carbons and COH glycerol carbons is split and the intensity of the glycerol signal at 63 ppm decreased due to release of some glycerol molecules from the starch structure making them more mobile.

Closer inspection on C1 and C4 resonances in the spectra for TPS55 and TPS-MMT55 samples reveals a change in the shape of C1 resonance and decrease in C4 resonance intensity after seven weeks of storage. This can be explained by ongoing recrystallization resulting in an increase in crystalline phase in the samples during storage at 55% RH. On the other hand, only minor changes were identified in the spectra for TPS85 and TPS-MMT85 samples stored for one and seven weeks, which indicates the structural stabilization of crystalline structure already after one week of storage at 85% RH. It can be suggested that an additional plasticizing effect of water resulting in enhanced starch chains mobility supports their arrangement in ordered structure, and thus it is responsible for rapid recrystallization during the first week.

### 3.2. ^1^H BL NMR

TPS immediately after preparation is a complex system consisting of starch chains in amorphous phase, plasticizer and water molecules, which may interact through hydrogen bonding with each other as well as with starch chains, or the chains can stay relatively free. Moreover, the distribution of glycerol and water molecules in the sample volume is inhomogeneous, so plasticizer-rich and starch-rich domains are formed resulting in different mobility of starch chains [27,28] as indicated by measured temperature dependences of DMTA loss factor tan δ discussed later. If TPS contains a small amount of nanofiller, the components of TPS can also interact with nanofiller particles. In the case of the investigated samples, glycerol, water and starch chains can be intercalated in the galleries of MMT particles or they may interact with the surface of exfoliated MMT.

BL ^1^H NMR spectra recorded on static polymer samples usually consist of one broad and one or several narrow lines whose widths reflect different mobility of hydrogen nuclei. The dominant mechanism of line broadening in ^1^H NMR spectra of starch-based materials are ^1^H-^1^H dipolar interactions which can be averaged by molecular motion. For this reason, broad and narrow lines in the spectra are assigned to hydrogen nuclei in rigid and more mobile domains, respectively. Samples stored in desiccators absorb different amounts of water from ambient atmosphere acting as an additional plasticizer [29,30] that influences overall mobility of ^1^H nuclei in the samples.

**Figure 3:**
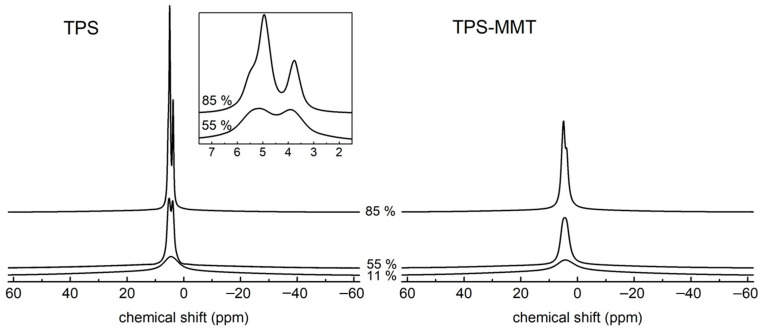
^1^H BL NMR spectra measured for TPS (**left**) and TPS-MMT (**right**) samples stored at indicated RHs for one week (all spectra are normalized according to the areas). The W, G(OH) and G(CH) signals relate to the hydrogens of water and glycerol OH and CH/CH_2_ groups, respectively.

The BL ^1^H NMR spectra for the studied TPS and TPS-MMT samples reflect the structural characteristics described above. Figure 3 shows their spectra measured after one week of storage at 11, 55 and 85% RH. The broad line relates to rigid starch chains located in both amorphous and crystalline regions and to a small amount of glycerol and water molecules entrapped in TPS structure if present. On the broad line, one or several narrow lines are superimposed; they are associated with water and glycerol molecules, and also with highly mobile starch chains if present in the sample.

In the spectrum for TPS11 sample only one structureless narrow line is observed pointing to very low mobility of TPS components. The narrow signal is superimposed on the broad line with full width at half maximum (FWHM) of ~25 kHz. Water content in this sample is very low—approximately 0.8 wt% as determined from weight measurements (discussed later)—and it is suggested that water contributes to the overall intensity in the spectrum only to a very small extent. One can expect that entrapped glycerol molecules in TPS structure are present in the sample contributing to broad signal in the spectrum. This fact was confirmed by deconvolution discussed later.

Spectra measured for the TPS samples stored at higher RH (Figure 3 left) differ significantly from the TPS11 spectrum, since two narrow signals appear in the spectra which can be assigned to overlapping signals produced by hydrogens of water (4.8 ppm) and glycerol (5.3 and 3.7 ppm). FWHM of the broad line is about 22 and 20 kHz in the TPS55 and TPS85 spectrum, respectively. As all samples originate from the same batch they can differ only in their water content. TPS55 and TPS85 samples absorbed significant amounts of water so that they contained ~11 and 28 wt% of water after one week, respectively (discussed later). Absorbed water had an additional plasticizing effect on TPS structure [29,30] leading to the increased mobility of all TPS components resulting in averaging of ^1^H-^1^H dipolar interactions. Therefore, TPS55 and TPS85 samples provide significantly narrower lines in the BL ^1^H NMR spectra compared to the TPS stored at 11% RH. The plasticizing effect of water manifested itself also by lowering the glass transition temperature (*T*_g_) of the starch-rich phase (DMTA results). It is seen that spectral lines measured for TPS-MMT samples are broadened when compared to relevant lines in TPS spectra (Figure 3 left). This fact can be explained by interactions of glycerol and water molecules and starch chains with MMT particles occurring in nanocomposite samples which hinder molecular motion of TPS components. This results in the decreased mobility and line-broadening; thus, the narrow line splitting is not observed.

**Figure 4:**
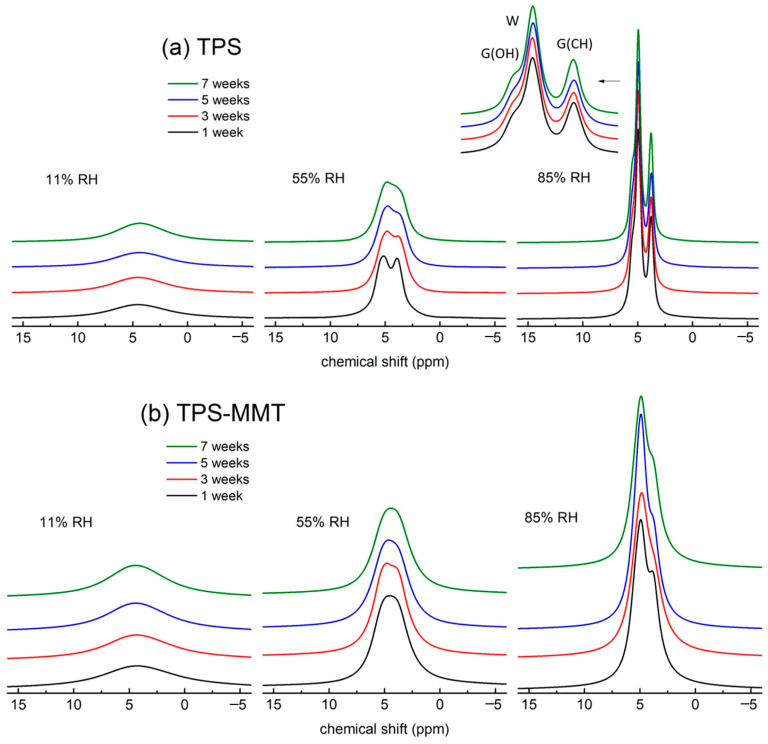
Central parts of the BL ^1^H NMR spectra for (**a**) TPS and (**b**) TPS-MMT samples stored at 11 (**left**), 55 (**middle**) and 85% (**right**) RHs measured after indicated storage time (spectra are normalized according to the areas; spectra (**b**) are enlarged twice with respect to spectra in (**a**)). The W, G(OH) and G(CH) signals correspond to the hydrogens of water and glycerol OH and CH/CH_2_ groups, respectively.

To assess the influence of long-term storage at various RHs, BL ^1^H NMR experiments of our samples were acquired throughout seven weeks (Figure 4). Only central parts of the spectra are presented for better clarity.

The shapes of the spectra for TPS11 and TPS-MMT11 samples (Figure 4a,b left) do not change significantly with time; they consist of one broad and one narrow line which keeps a structureless character. The width of the broad line remains approximately the same in all spectra pointing to the fact that mobility of rigid domains did not change during storage of the samples in the desiccator. On the contrary, the width of the narrow line decreased with storage time. This can be explained by physical ageing of the samples occurring at temperatures below *T*_g_ in starch-rich regions [31,32], during which the starch structure approaches thermodynamic equilibrium, free volume increases and glycerol and water molecules acquire higher mobility. DMTA measurements for both TPS and TPS-MMT samples stored at 11% RH showed that *T*_g_ of starch-rich phase for these samples is ~70 °C confirming they occur under *T*_g_ and physical ageing could take place. Crystalline structure does not form under *T*_g_, which is in accordance with the results obtained from ^13^C CP MAS NMR spectra (Figure 1).

The BL ^1^H NMR spectra for TPS stored at 55% RH are shown in Figure 4a, middle. The spectrum resolved in two lines was obtained after one week of storage when the sample already underwent recrystallization as observed from ^13^C NMR spectra and also reached equilibrium water content (discussed in Section 3.4). Recrystallization during the first week caused free volume increase in amorphous TPS domains and thus higher mobility of water and glycerol molecules. With longer storage time narrow lines broadened, which resulted in less resolved spectra. This fact can be explained by migration of the water and glycerol molecules in amorphous TPS domains making possible interactions between each other and/or with starch chains which decreased their mobility and enlarged their chemical shifts distribution. Formation of these hydrogen bonds makes the structure stiffer, and both tensile stress and Young’s modulus increased while tensile strain decreased after seven weeks of storage (as discussed later). Similar features are observed also for the spectra measured for nanocomposite stored at 55% RH (Figure 4b middle).

The best resolved ^1^H BL NMR spectra were detected for the TPS85 sample (Figure 4a right) due to the highest amount of absorbed water causing very high mobility of all TPS components. Concerning structural behavior in time, the widths of narrow lines slightly decreased after seven weeks of storage which is in contrast with TPS55 sample (the narrow lines broadened). The samples stored at 85% RH absorb water up to five weeks of storage (discussed in Section 3.4). During this period water molecules penetrate into an amorphous phase of the TPS structure that leads to a reduction in starch intermolecular hydrogen bond density. As a result, certain deterioration of mechanical properties was observed; tensile stress and Young’s modulus decreased. Moreover, during penetration of water molecules into TPS structure, regions rich in glycerol or water could be formed and thus these molecules along with starch chains acquired higher mobility. This explains the decrease in narrow lines’ widths for the TPS85 sample. Similar structural changes as took place in the TPS85 sample can be expected to occur in TPS-MMT85 sample as well; however, phase separation might be hindered by additional interactions of water and glycerol molecules and starch chains with nanofiller. This is confirmed by the fact that narrow lines widths did not change with time as deduced from BL ^1^H NMR spectra (Figure 4b right).

In summary, we can say that high water content in the samples stored at 85% RH caused reduction in starch intermolecular H-bond density after seven weeks of storage. A similar effect was reported by Leroy et al. [33] who analyzed thermal and structural (WAXS) behavior of thermoplastic potato starch stored under 58 and 89% RH.

### 3.3. ^1^H MAS NMR

Additional information concerning molecular mobility in the studied samples can be obtained using magic angle spinning (MAS) technique. This technique averages dipolar interactions which results in high-resolution solid-state NMR spectra. However, relatively narrow signals accompanied by spinning sidebands at multiples of the MAS rate can be still superimposed on a broad signal [26].

**Figure 5:**
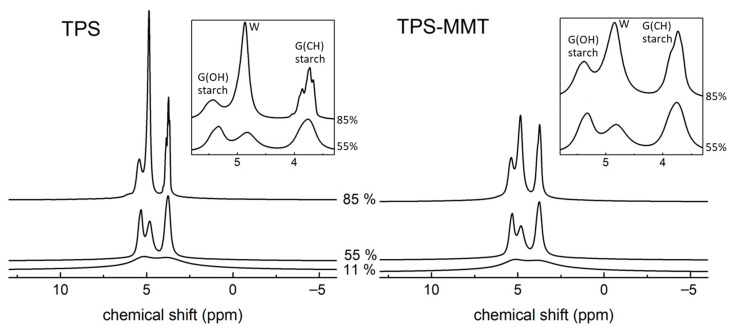
MAS ^1^H NMR spectra measured for TPS (**left**) and TPS-MMT (**right**) samples stored one week at indicated RHs (all spectra are normalized according to the areas). The W, G(OH) and G(CH) signals correspond to the hydrogens of water and glycerol OH and CH/CH_2_ groups, respectively; starch hydrogens signals overlap with G(OH) and G(CH) signals.

Figure 5 shows ^1^H MAS NMR spectra measured for TPS and TPS-MMT samples stored for 1 week at 11, 55 and 85% RHs; one can notice that the spectra are significantly better resolved than corresponding BL ^1^H NMR spectra (Figure 3). The peaks in these spectra are related to hydrogens of water, glycerol and starch chains. Despite using the MAS technique, the spectra for TPS11 and TPS-MMT11 samples show only signs of splitting in two lines with positions at 5.1 and 3.8 ppm. Better resolution was obtained in the TPS55 and TPS-MMT55 spectra indicating increased mobility of TPS components when the water content is higher. The spectral line at 5.1 ppm is split into two well-resolved lines with positions at ~5.3 and 4.8 ppm related to glycerol OH groups and water, respectively. The line at 3.8 ppm is related to glycerol CH and CH_2_ groups [20]. The best resolution was obtained in the TPS85 spectrum indicating the highest mobility of TPS components due to the highest water content. Moreover, signal at 3.8 ppm shows splitting in several lines associated with starch hydrogen nuclei [20,34]. In the TPS-MMT85 spectrum this line is prevented from splitting due to additional interactions between starch chains and nanofiller. Highly mobile starch hydrogen nuclei form a signal at position 5.3 ppm in addition to signals between 3 and 4 ppm; however, the former signal is overlapped with the signal related to glycerol OH groups. It is possible that also in the case of TPS55 and TPS-MMT55 samples, highly mobile starch chains are present within the structure contributing to 5.3 and 3.8 ppm signals, but they are not resolved in the ^1^H MAS NMR spectra. Their presence will be confirmed by deconvolution of the BL ^1^H NMR spectra discussed later.

The linewidths of the signals in the spectra for TPS11 and TPS-MMT11 samples decreased with time, which could be explained by physical ageing as it was discussed in BL section. ^1^H MAS NMR spectra for the TPS55, TPS-MMT55 and TPS85 samples show only negligible differences when measured after various storage times in contrast to BL spectra due to the effect of MAS averaging dipolar interactions. On the other hand, in the case of the TPS-MMT85 sample the signals at 5.3 and 3.8 ppm slightly broadened after seven weeks as a result of newly emerging interactions between glycerol and starch hydrogens with MMT particles. This is in accordance with results obtained from BL spectra.

### 3.4. Water Content Estimation by Means of BL ^1^H NMR

The starch-based materials are very sensitive to storage conditions, especially to relative humidity, because starch can easily uptake water from ambient [35]. The water content then strongly influences the structure, recrystallization, and mechanical properties of these materials [33,36,37]. In TPS samples, mobile molecules of water denoted as free water and immobilised water molecules create strong bonds with starch chains [20,38] and present in MMT interlayer galleries termed bound water can be found. The well-defined ratios of starch, glycerol, water and montmorillonite (in the case of nanocomposite) used in the preparation process of TPS and nanocomposite samples directly offer mass ratio of starch and glycerol which remains unchanged during preparation, after drying or during storage. However, this does not apply to water, because its amount changes during the above-mentioned processes.

Deconvolutions of the BL ^1^H NMR spectra provide intensities of deconvoluted lines which are proportional to the number of ^1^H nuclei associated with particular lines. This fact allows the estimation of free water content in some cases.

The BL ^1^H NMR spectra for TPS11 and TPS-MMT11 samples were deconvoluted in one broad and one narrow line at chemical shift of 5.5 and 4.4 ppm, respectively. It can be expected that due to the very low amount of water in these samples, the contribution of water hydrogens to the signals is negligible and thus only hydrogens of starch and glycerol form the signals. The deconvolutions provide broad to narrow lines intensity ratio of ~1.6, which is higher than theoretical starch/glycerol hydrogen ratio equal to 1.42 derived from composition of the sample. This fact can be explained by the presence of a certain portion of glycerol molecules immobilized in the TPS structure which contributes to broad signal. It was impossible to estimate water content from these deconvolutions.

The water content estimation was carried out for TPS and nanocomposite samples stored at 55 and 85% RHs. First, their BL ^1^H NMR spectra were deconvoluted in one broad line related to hydrogen nuclei located in rigid starch chains and bound water molecules, and three narrow lines at chemical shifts of ~5.3, 4.8 and 3.8 ppm whose position was derived from MAS ^1^H NMR spectra (Figure 5); free water hydrogens form signal at 4.8 ppm, glycerol OH groups at 5.3 ppm, glycerol CH and CH_2_ groups at 3.8 ppm, and hydrogen nuclei in mobile starch chains at 5.3 and between 3 and 4 ppm [20,34]. Signal of the mobile starch chains might thus overlap with glycerol signal in our spectra. Indeed, the deconvolutions of the spectra provide the intensity ratio of broad line to narrow lines at 5.4 and 3.9 ppm approximately 1 which is lower than theoretical starch/glycerol hydrogen ratio of 1.42. Thus, we assume that at high RH (55 and 85%), the plasticizing effect of water on TPS structure resulted in high mobility of a certain fraction of starch chains comparable with mobility of water and glycerol molecules [28], since their signals appear in the spectra and overlap with glycerol signals.

**Figure 6:**
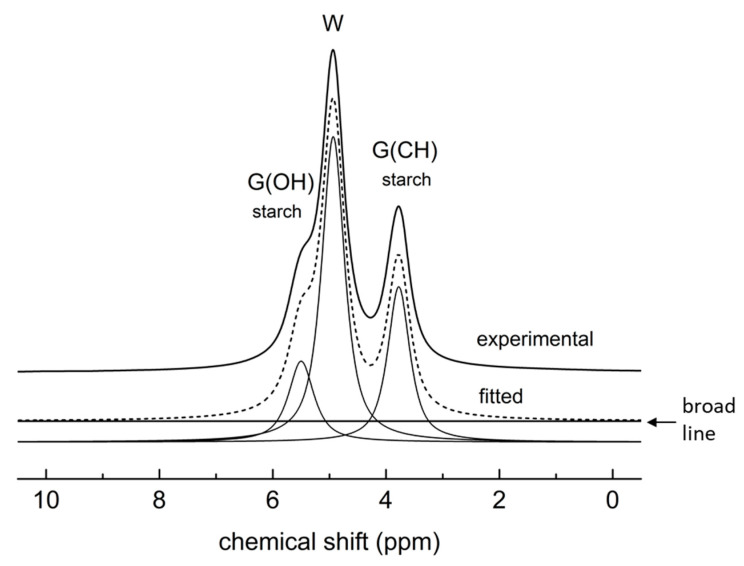
Deconvolution of the BL ^1^H NMR spectrum for TPS85 sample stored seven weeks to one broad and three narrow lines. The W, G(OH) and G(CH) signals correspond to the hydrogens of water and glycerol OH and CH/CH_2_ groups, respectively; starch hydrogens signals overlap with G(OH) and G(CH) signals.

**Figure 7:**
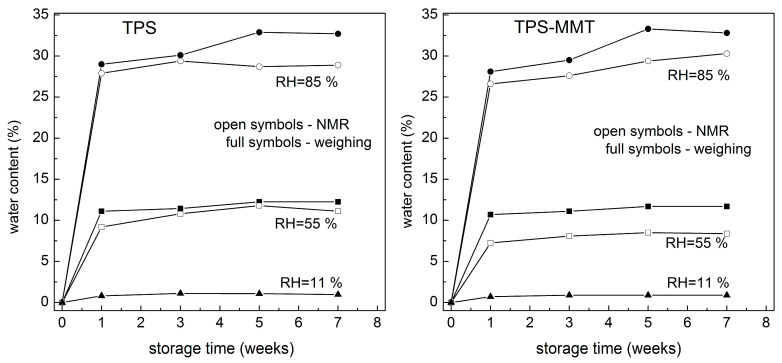
Dependence of water content on storage time at 11, 55 and 85% RHs for TPS and TPS-MMT samples based on their weighing (total water content) and estimated from BL ^1^H NMR analysis (free water content).

The deconvolution for the TPS85 sample stored seven weeks is shown in Figure 6 as an example; intensities of 0.117, 0.380 and 0.192 related to narrow lines at 5.3, 4.8 and 3.8 ppm were obtained. The intensity of the broad line was 0.311. Based on this determination and knowing the theoretical starch/glycerol hydrogen ratio of 1.42, the relative amount of free water in the sample was calculated. The fact that a certain fraction of starch hydrogen nuclei contributes to narrow lines at 5.3 and 3.8 ppm was also considered. Estimated free water content by means of BL ^1^H NMR for TPS and nanocomposite samples during storage at 55 and 85% RH is shown in Figure 7 along with water content determined from weighing of the samples. The most significant increase in water absorption was observed during the first week of storage for all samples. This was expected as the samples were dried before storage. During the following weeks the water content in TPS55 and TPS-MMT55 samples increased only slightly, but the increase in the samples stored at 85% RH was significant up to five weeks and then levelled off. Water content estimated by deconvolution of BL ^1^H NMR spectra is systematically lower than that evaluated by weighing the samples, since only free water can be detected using the NMR technique in contrast to weighing, which allows estimation of total water content in the sample. It is in accordance with assumption that “free” and “bound” water is present in the structure. Moreover, a greater difference in estimation of water content by mentioned methods for nanocomposite samples reveals a lower amount of free water in these samples because water molecules can be intercalated in the galleries of MMT particles, or they interact with the surface of exfoliated MMT.

### 3.5. Mechanical Properties

Mechanical properties are considered to be important parameters concerning TPS performance. These properties depend significantly on surrounding relative humidity and storage time. The values of ultimate tensile strength, elongation at break, and Young’s modulus for TPS and TPS-MMT samples stored at various humidities for one and seven weeks are shown in Table 1. It is seen that the tensile strength and Young’s modulus of the dried samples and the samples stored at 11% RH increase with the incorporation of MMT. This increase is ascribed to the formation of hydrogen bonds between starch chains, water, glycerol, and hydroxyl groups of MMT layers [3,39]. Concerning the relation of tensile strength changes with the humidity, it is seen that the tensile strength of both TPS and TPS-MMT samples is decreasing with the rising RH, while the tensile strength of the dried samples and the samples stored at 11% RH are identical. Moreover, the increase in RH results in a substantial decrease in Young’s modulus compared to the dried samples. The higher amount of the absorbed water at higher RH has an additional plasticizing effect, leading to an increase in the mobility of starch chains. However, lower RH will not affect the structure. Therefore, these findings indicate the relationship between tensile strength, Young’s modulus, and surrounding RH. As expected, the increase in RH and consequently increased chain mobility led to the increase in elongation at break. Similar behavior is observed for both TPS and TPS-MMT samples.

Regarding the aging effect on mechanical properties, the samples stored at 11% RH showed a decrease in tensile strength and Young’s modulus, while elongation at break increased which might be caused by physical ageing of the samples as discussed in ^1^H BL NMR results when glycerol and water molecules acquire higher mobility. The TPS samples stored at 55% RH showed a slight increase in tensile strength and Young’s modulus while the elongation at break decreased. This phenomenon is ascribed to a formation of new interactions between starch, glycerol, and water after reaching the equilibrium moisture content, resulting in the formation of hydrogen bonds and thus a slight increase in tensile strength and Young’s modulus. On the other hand, the TPS samples stored at 85% RH presented a decrease in all mechanical properties. Penetration of a high amount of water into the TPS structure followed by reduction in hydrogen bond density can be considered as the main reason to explain the decrease in mechanical properties compared to 55% RH. All these results are supported by NMR spectra.

### 3.6. Dynamic Mechanical Thermal Analysis (DMTA)

**Figure 8:**
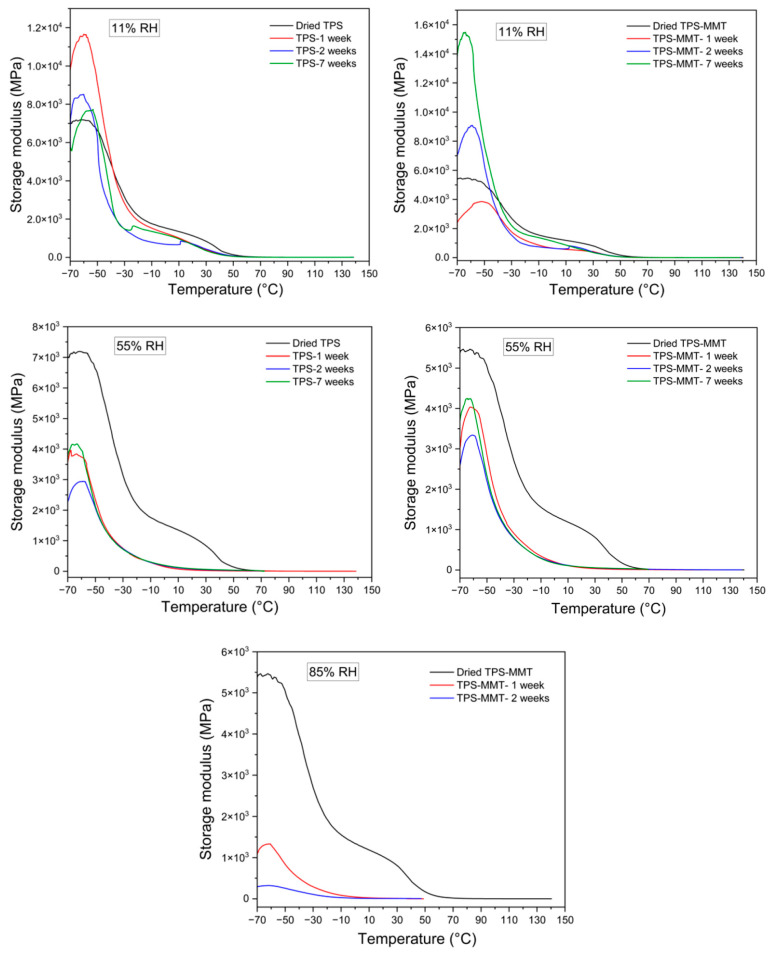
Storage modulus curves for TPS and TPS-MMT samples stored at 11, 55 and 85% RHs for one, two, and seven weeks of storage.

**Figure 9:**
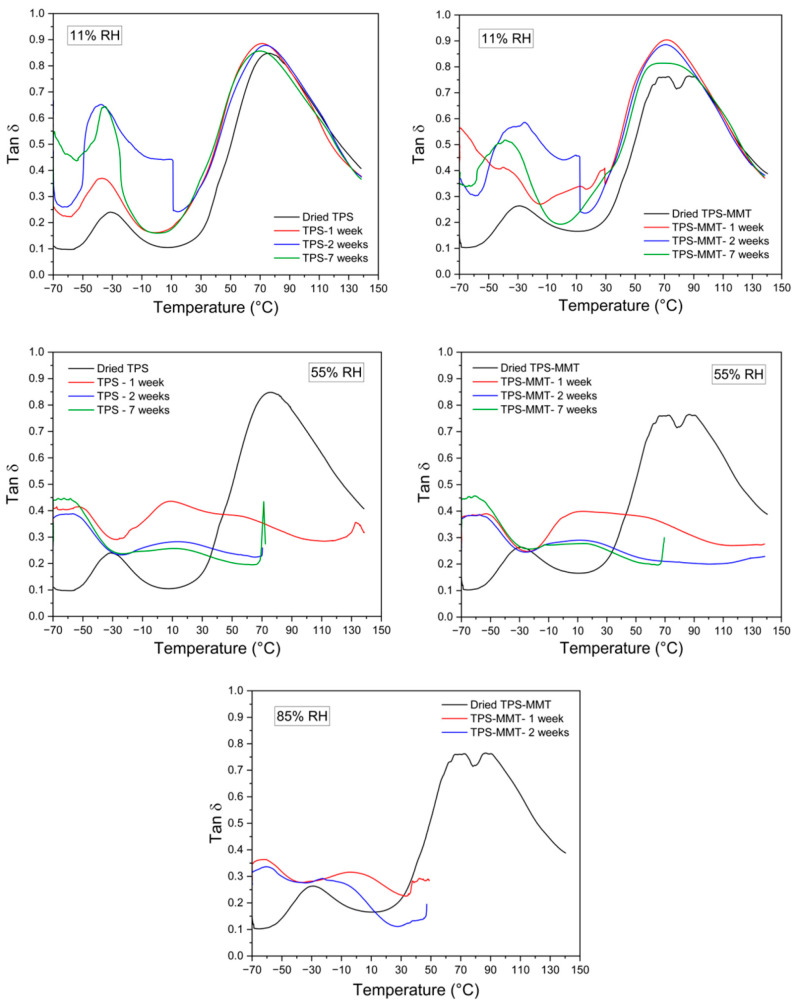
Tan δ curves for TPS and TPS-MMT samples stored at 11, 55 and 85% RHs for one, two, and seven weeks of storage.

DMTA measurements were performed to evaluate the viscoelastic response of the TPS samples, which is associated with the macromolecular motions in the structure. The storage modulus and tan δ of the TPS and TPS-MMT samples stored at various humidities for different times of storage are shown in Figure 8 and Figure 9, respectively. As expected, the storage modulus of the TPS samples increases with the incorporation of MMT particles indicating reinforcement effects of the MMT layers. Moreover, it can be seen that the storage modulus of the samples decreases with the rising RH. This result is attributed to the plasticizing effect of water molecules. Tan δ as a function of temperature for TPS samples indicated two relaxation peaks. The low-temperature relaxation peak corresponds to the glass transition of glycerol-rich domains, while the high-temperature peak is ascribed to the starch-rich domains [1,39]. The temperatures of maxima tan δ determined from curves in Figure 9 are summarized in Table 2. As expected, *T*_g_ values for TPS-MMT nanocomposites are higher than for TPS samples due to the restrictions in starch chain mobility caused by a certain extent of intercalation of plasticizer molecules and starch chains into the MMT platelet galleries. Furthermore, as seen in Table 2, the first peak, which is controlled by the plasticizer molecular motion, showed a shift to lower temperature for samples stored at higher RH, indicating that regions rich in glycerol or water might be formed during water absorption. On the other hand, samples stored at 11% RH exhibited a slight decrease in *T*_g_ compared to the dried samples, whereas the *T*_g_ values for the samples stored at 55% RH decreased substantially. The presence of more water may decrease the intermolecular hydrogen bond density [33]. However, low water content could not significantly affect the starch chain mobility, leading to preventing recrystallization during storage. It should be noted that measuring the DMTA for the samples stored at 85% RH was possible only for the TPS-MMT samples stored for two weeks or less. After maintaining the TPS samples longer at high humidity, the material structure is too soft to be fastened in the instrument clamps since either the samples are broken during fastening before starting the measurement, or the sample is slipping inside the clamp so that the length of the measured sample is not constant.

## 4. Conclusions

The effect of storage at various relative humidities on the structure, molecular mobility and mechanical properties of TPS and its nanocomposite was studied. It was shown that the amount of water has a crucial influence on all measured properties. Water content of the samples was determined from the weight measurements and by means of deconvolution of BL ^1^H NMR spectra, where the latter provided systematically lower values since only free water can be distinguished in the spectra.

Both TPS and nanocomposite samples stored at 11% RH at room temperature were brittle and preserved their glassy state. They did not recrystallize due to the small amount of water and for this reason only physical ageing could take place throughout the whole period of storage. On the contrary, the samples stored at higher RHs (55 and 85%) absorbed a significant amount of water acting as a plasticizer and lowering their *T*_g_ under room temperature which allowed the samples to recrystallize during the first week of storage. After seven weeks of storage the samples stored at 55% RH showed improvements in mechanical properties (tensile strength and Young’s modulus increased) due to newly formed interactions between starch, glycerol, and water making the structure stiffer. On the other hand, during storage at the highest RH (85%) the water molecules significantly reduced starch intermolecular hydrogen bonding density leading to deterioration of all mechanical properties measured.

The reinforcing effect of MMT nanoparticles manifested itself by improving the mechanical properties of nanocomposite samples when compared to TPS samples and led to lower molecular mobility of the TPS components. From the point of view of the material final application in practice, we could conclude that the best performance of the studied TPS-based nanocomposite is reached when stored at 55% RH.

## Figures and Tables

**Table 1:** Mechanical properties, namely tensile strength, elongation at break and Young’s modulus of the TPS and TPS-MMT samples stored at various humidities for one and seven weeks.

	Sample Code	Dried	11% RH		55% RH		85% RH	
			1 Week	7 Weeks	1 Week	7 Weeks	1 Week	7 Weeks
Tensile Strength (MPa)	TPS	7.8 ± 2.7	9.3 ± 0.8	6.8 ± 0.5	1.3 ± 0.0	2.5 ± 0.1	1.3 ± 0.0	0.6 ± 0.1
	TPS-MMT	10.4 ± 1.1	11.5 ± 1.0	7.6 ± 0.2	1.4 ± 0.1	2.4 ± 0.4	1.3 ± 0.1	0.6 ± 0.1
Elongation at break (%)	TPS	1.5 ± 0.3	4.3 ± 1.6	27.1 ± 11.5	66.6 ± 2.1	34.4 ± 2.0	25.3 ± 1.2	8.2 ± 0.4
	TPS-MMT	1.7 ± 0.2	3.7 ± 0.8	31.4 ± 5.0	64.1± 8.9	27.8 ± 5.5	24.7 ± 1.1	7.6 ± 1.3
Young’s Modulus (MPa)	TPS	970.2 ± 207.5	662.9 ± 74.7	297.4 ± 2.4	9.2 ± 0.5	25.2 ± 3.4	10.5 ± 0.4	9.4 ± 0.8
	TPS-MMT	1148.2 ± 47.4	703.0 ± 68.9	435.9 ± 4.3	10.0 ± 1.1	26.5 ± 1.3	10.9 ± 0.6	9.7 ± 0.5

**Table 2:** Temperatures of maximum values for tan δ appearance as extracted from Figure 9.

Sample	1st Peak T (°C)				2nd Peak T (°C)			
	Dried	11% RH	55% RH	85% RH	Dried	11% RH	55% RH	85% RH
TPS	−31.3				75.5			
TPS-MMT	−28.9				86.7			
TPS- 1 week		−37.1	−54.0	n/a		71.3	8.9	n/a
TPS- 2 weeks		−37.6	−56.6	n/a		74.0	14.1	n/a
TPS- 3 weeks		−37.0	−56.0	n/a		72.4	6.1	n/a
TPS- 5 weeks		−35.6	−53.2	n/a		71.2	20.2	n/a
TPS- 7 weeks		−35.2	−58.5	n/a		69.9	11.3	n/a
TPS-MMT- 1 week		−40.2	−52.9	−61.8		70.9	13.3	−4.2
TPS-MMT- 2 weeks		−32.5	−57.5	−60.6		70.7	11.6	−22.6
TPS-MMT- 3 weeks		−20.3	−57.7	n/a		64.0	10.2	n/a
TPS-MMT- 5 weeks		−33.3	−55.4	n/a		72.7	2.8	n/a
TPS-MMT- 7 weeks		−38.5	−60.6	n/a		69.8	13.6	n/a

## Data Availability

The data presented in this study cannot be shared at this time as they form a part of an ongoing research.
